# Re-examining the robustness of voice features in predicting depression: Compared with baseline of confounders

**DOI:** 10.1371/journal.pone.0218172

**Published:** 2019-06-20

**Authors:** Wei Pan, Jonathan Flint, Liat Shenhav, Tianli Liu, Mingming Liu, Bin Hu, Tingshao Zhu

**Affiliations:** 1 Institute of Psychology, Chinese Academy of Sciences, Beijing, China; 2 University of Chinese Academy of Sciences, Beijing, China; 3 Center for Neurobehavioral Genetics, Semel Institute for Neuroscience and Human Behavior, University of California Los Angeles, Los Angeles, United States of America; 4 Department of Computer Science, University of California Los Angeles, Los Angeles, United States of America; 5 Institute of Population Research, Peking University, Beijing, China; 6 School of Information Science & Engineering, Lanzhou University, Lanzhou, China; National Institutes of Health, UNITED STATES

## Abstract

A large proportion of Depression Disorder patients do not receive an effective diagnosis, which makes it necessary to find a more objective assessment to facilitate a more rapid and accurate diagnosis of depression. Speech data is easy to acquire clinically, its association with depression has been studied, although the actual predictive effect of voice features has not been examined. Thus, we do not have a general understanding of the extent to which voice features contribute to the identification of depression. In this study, we investigated the significance of the association between voice features and depression using binary logistic regression, and the actual classification effect of voice features on depression was re-examined through classification modeling. Nearly 1000 Chinese females participated in this study. Several different datasets was included as test set. We found that 4 voice features (PC1, PC6, PC17, PC24, *P*<0.05, corrected) made significant contribution to depression, and that the contribution effect of the voice features alone reached 35.65% (*Nagelkerke's R*^*2*^). In classification modeling, voice data based model has consistently higher predicting accuracy(F-measure) than the baseline model of demographic data when tested on different datasets, even across different emotion context. F-measure of voice features alone reached 81%, consistent with existing data. These results demonstrate that voice features are effective in predicting depression and indicate that more sophisticated models based on voice features can be built to help in clinical diagnosis.

## Introduction

Depression disorder is the commonest psychiatric disorder (the lifetime prevalence reaching 16.2%) [1–7] and is now the leading cause of disability [8]. Yet despite its prevalence, many cases of depression remain unrecognized [9], depriving many of the possibility of receiving effective treatment. Fewer than half of those eligible receive treatment and in many countries the figure is less than 10% [10]. One of the main obstacles in the way of treatment provision is the difficulty of recognizing and diagnosing depression. Diagnosis currently requires interview by a clinician, often over half an hour or more, a method that rarely exceeds an inter-rater reliability of 0.7 (kappa coefficient) [11]; in one large field study reliability was estimated to be as low as 0.25 [12]. According to a meta-analysis of 41 studies [13], community doctors had an accuracy of 47% in recognizing patients with depression recognition accuracy of depression by general practitioners is 47.3%. Methods to identify cases of depression that can be deployed at an appropriate scale are needed.

Researchers have attempted to find objective methods to increase the accuracy of depression diagnosis. Blood transcriptomic biomarkers and acoustic biomarkers, among other methods, have been investigated to help detect depression [14,15]. Among these, voice analysis has attracted increasing attention, being easy-to-acquire, non-invasive, and having objective advantages.

The voices of depression patients have recognizable characteristics, including slow speech rate, frequent pauses, little difference in speech features, and lack of cadence [16]. Voice features have been used in machine-learning methods to help diagnose depression. Multiple acoustic features have been investigated in empirical studies, including spectral, cepstral, prosodic, glottal, and Teager energy operator features [17–21] Potentially confounding factors may challenge the validity of such published studies, however.

The impact of confounding factors has been pervasively neglected in most of the current research on this topic. It has been suggested by many studies that demographic variables are significantly associated with depression. For example, occupations with higher status protect against depressive symptoms, and workers in specific occupational sectors and types report different levels of depressive symptoms [22]. What is more, demographic factors can also impact individual voice features, raising concerns [23]. For instance, the fundamental frequency (voice pitch) of females is usually higher than that of males [24]. Other studies have mentioned that demographic factors, such as age, gender, emotion, and the characteristics of the speaker can impact the classification results [25–27].

Several studies have attempted to explain the existence of nuisance factors and to control these to a certain degree. Sex-independent classifiers have been suggested, and have achieved better results [19,20,28,29]. However, none of these studies have examined the extent to which demographic information contributes to depression prediction. More importantly, the association between voice features and depression has not been evaluated to a significant level of confidence, and there has not been any reference to compare the predictive effect of voice features to. In addition, sample sizes in all studies are relatively small limiting power to detect subtle but possibly important classifiers [30–32], especially when there are some subcategories, such as gender.

In this study, the primary aims were: 1) to evaluate whether voice features can significantly contribute to the prediction of depression; 2) replicate the previous classification results for voice features on depression and examine the generalization ability of classification models; and 3) identify the effect of voice features and compare this to demographic variables. Once voice features have been shown to be useful in predicting depression, future research can confidently explore more robust classification models to aid in clinical depression diagnosis.

## Materials and methods

### Data collection

We have two different data sets. The first one is used for building models, named interview speech dataset. The second one is only for model test, which named 973 dataset. They are from different project with different research project.

#### Interview speech dataset

In this dataset, all subjects were interviewed using a computerized assessment system, during which participants’ voice was recored. All participants were Chinese females with Han nationality and had three generations of Han nationality relatives. The depression patients (the case group) were all diagnosed by psychiatric specialists with DSM-5. All had two or more major depression episodes. Comorbidities, such as bipolar disorder and other mental deficiencies, were excluded. The healthy participants (the control group) had no experience of depression or any other mental illnesses. There were no blood relationships between the two groups. The research design is described in detail in a previous report [33, 34]. All participants were between 30 and 60 years old.

Only the voice datasets from the demographic questions numbered D2.A and D2.B were chosen for analysis because the others were either too short or the sample size was too small. Question D2.A is *When is your birthday*? The voice recordings of its answer varies from 0.29 to 33.09 s. Question D2.B is *How old are you*? The voice recordings of its answer ranges from 0.11 to 11.78 s. In this research, voice recordings of question D2.A was used to build logistic regression equations and classification models. While voice recordings of question D2.B was used as a test set for classification models mentioned above, as it has high homogeneity with D2.A dataset but not exactly the same—not all participants has both voice recordings for D2.A and D2.B. For convenience, these two datasets will be named as D2.A, D2.B.

It should be noted that each participant provided one voice sample, and all voice files were saved in a .wav/16 bit/16kHz format.

The study protocol was approved centrally by the Ethical Review Board of Oxford University (Oxford Tropical Research Ethics Committee) and the ethics committees of all participating hospitals in China. All participants provided their written informed consent.

#### 973 dataset

This dataset was collected from the 973 project [35].

In this project, there was a priming of different emotions(positive, neutral, negative), under which participants’ voice recordings were collected by different tasks(video watching, text reading, interview, question answering, picture describing). Participants from case group is diagnosed by clinicians with DSM-IV, their depression severity varies. For the control group, participants are both physically and mentally healthy people. Each participant experienced all three emotion priming three different emotion priming. 73 participants were included, 34 healthy individuals and 39 depressed patients. Their age also ranges between 30–60 years old. This dataset was included in this research to investigate whether models built by interview speech dataset can be used to predict voice recordings from different emotion context.

### Data preprocessing

#### Interview speech dataset

After the data was cleaned (clearly audible, clips of doctors’ voices cut), we chose sample for which complete demographic information (six demographic variables: age, accent, education, occupation, marital status, social class) was available. There were 1132 participants (584 depression patients and 548 healthy people) for question D2.A, and 904 participants (500 depression patients and 404 healthy people) for D2.B.

There are 6 different demographic variables in this dataset: age(30~60), occupation(1work; 2 wait for employment; 3 retired; 4 housewife; 5 others), education(1 uneducated; 2 primary school; 3 junior high school; 4 senior high school; 5 junior college; 6 senior college; 7 college and 8 above college), marital.status(1 married; 2 living apart; 3 divorced; 4 widowed; 5 single), social.class and accent. The accent areas were classified based on city and province information (Institute of Linguistics, CASS, 2012). There are 15 accent areas involved in our dataset: Beijing Mandarin, Zhongyuan Mandarin, Dongbei Mandarin, Jianghuai Mandarin, Lanyin Mandarin, Jilu Mandarin, Jiaoliao Mandarin, Xinan Mandarin, Gan, Xiang, Jin, Wu, Min, Yue, and Tianjin. This variable was then translated into a dummy variable for further analysis.

At this point, each sample had one column headed with an ID number, 988 columns of voice feature data, six columns of demographic data, and one column containing the depression diagnosis. The dependent variable was depression and was divided into two classes: depressed (labeled 1) or not (labeled 0).

For feature extraction, based on existing findings, we chose 26 physical features that have been widely used in emotion recognition [36–38]: intensity, loudness, zero-crossing rate, voicing probability, fundamental frequency (F0), F0 envelope, eight line spectral pairs (LSP), and 12 mel-frequency cepstral coefficients (MFCC). The delta values, which reflect dynamic change in voice features, were then calculated for each of these 26 static features, and also for 19 statistical features, including maximum, minimum, range, mean, standard deviation, skewness, etc. By employing openSMILE [39], a total of 988 voice features were obtained [40] The voice data was then standardized.

#### 973 dataset

For 973 dataset, all voice recordings were complete, no need to cut. And the background noise was well-controlled in this project. 988 voice features were extracted in the same way as interview data.

For 973 dataset, the demographic variables are age, education and occupation.

### Data description

Most of the demographic variables were significantly different between case and control group in D2.A, D2.B and for 973 project. As some of the sample size for a category is too small, we performed all difference test based on the permutation test. To simplify the content and highlight the most important analysis in this research, we put all tables about data description as S1 Table in *Supporting Information*. As there are many biases between cases and controls in demographic variables, we need to match cases and controls to control the biases in demographic variables as much as we can.

### Confounders matching

Propensity score matching (PSM) is a statistical matching technique that attempts to estimate the effect of a treatment, policy, or other intervention by accounting for the covariates that predict receiving the treatment. PSM attempts to reduce the bias due to confounding variables that could be found in an estimate of the treatment effect obtained from simply comparing outcomes among units that received the treatment versus those that did not [41]. We used matchIt package from R to match cases and controls on those confounders—demographic variables.

### Data description after matching

After propensity score matching, most of the difference tests were changed to insignificant, or its z value, chi-square value decreased. But it should be noted that there are still many demographic variables significantly differed between groups. That is to say, our data is still biased to some level. Please see S1 Table in the *Supporting Information*.

### Data analysis

#### Binary logistic regression

As our data is biased, we need to check the contribution of demographic variables and voice data separately. And compare model fitness when using voice data alone, demographic data alone, and two combined(voice data + demographic data) predicting depression status to separate out the effect of voice data. Hence in the first stage, we employed statistical methods to explore whether voice data can significantly predict depression. We built three logistic regression models described above to investigate the contributing effect of both voice features and demographic variables when predicting depression. And we used ANOVA test to compare model fitness among these models. These analyses were based on dataset D2.A.

Principal component analysis (PCA) was initially conducted in order to reduce data dimension and avoid the multicollinearity problem. First, only demographic variables entered model. Second, only voice feature data entered model. Third, voice data and demographic data entered logistic model together.

Both the odds ratio (*OR*) and *Nagelkerke's R*^*2*^ were employed as indicators of the contributing effects of the variables to depression. The *OR* [42] is the exponent based on the natural constant, *e*, which explains the extent to which the change in the independent variable causes the change in probability of the dependent variable. When the *OR* value is >1, the corresponding variable is the risk factor for depression; higher values imply a greater contributing effect to the dependent variable. *Nagelkerke's R*^*2*^ [42] equals the adjusted *R*^*2*^ in linear regression, which also means that the value of *Nagelkerke's R*^*2*^ provides the amount of variance of the dependent variable explained by the explanatory variable. The stratified entry method allowed us to examine the contributions of important variables of interest gained after controlling the confounding variables.

#### Classification modeling

In the second stage, we tested the actual classification effect of voice features on depression using supervised learning methods. We built several classification models with identical data from question D2.A.

We split D2.A into training set and test set with ratio 7:3. All classification models were built based on the 70% training set of D2.A. To investigate predicting effect of voice data, we take classification models built on demographic variables as baseline to compare. To test model robustness, we tested the classification models on three different test sets. The first one is the rest 30% voice data of D2.A. The second one is the D2.B dataset. The third one is the 973 dataset. It should be noted that to investigate whether the models we built have robust predicting effect under different emotion context, we split 973 dataset as three test sets: data set under positive, neutral and negative emotion context separately.

The voice data were high dimensional, i.e. many variables were measured. To improve the generalizability of the models and to avoid the curse of dimensionality, feature selection was implemented, using random forest as the selection strategy with Boruta package in R [43].

The depression variable (labeled as 0 and 1) was included as a classification label. Classification models were built based on the training set, then the model performance was assessed on the test set. The classification models were built using random forest [44]. With each test set, three models were built, having only demographic data input, only voice data input, and both voice data and demographic data input, respectively, in order to compare the classification results under different situations. We used those identical models to select the same features under question D2.B and three 973 datasets, then classified them to estimate the generalization abilities of the models built on the training set of D2.A.

*F measure* [45], an evaluation indicator, was employed to assess the accuracy of the classification models. It considered both the precision) and the recall) of the classification models in order to compute the score. Precision is the number of correct positive results divided by the number of all positive results returned by the classifier. Recall is the number of all samples that should have been identified as positive, divided by the number of correct positive results returned by the classifier.

## Results

### Binary logistic regression

We examined how much demographic variables and voice features contributed to depression, using *Nagelkerke's R*^*2*^ statistic, and estimated the effect of each variable using *OR*s. More importantly, we also compared model fitness among different variable input. The results showed that when only demographic data in model, it accounted for 10.87% (*Nagelkerke's R*^*2*^) of the variance in the dependent variable depression. *OR*s for each demographic variable and their significance are shown in Table 1; age (*OR* = 0.95, *P<0*.*0001*), occupation (*OR* = 1.17, *P*<0.0001), and wu accent (*OR* = 2.90, *P* = 0.019) significantly predicted depression.

**Table 1:** Binary logistic regression model of demographic data.

	*β*	SE	*Wald*	*corrected P*	*OR*
**age**	-0.05	0.01	-4.83	2.1E-04***	0.95
**occupation**	0.15	0.04	4.07	7.4E-03**	1.17
**wu**	1.07	0.24	4.43	1.5E-03**	2.90

*P*<0.01**,

*P*<0.001***;

Bonferroni correction

We applied PCA to the 988 voice features in order to reduce the data. We found that 137 principal components (PCs) captured 90% of the original data variance, with the PCs showing no significant correlation in a pairwise correlation analysis.

When only voice data entered logistic model, it accounted for 35.65% of variance in the dependent variable depression(Nagelkerke’s R^2^). ORs for each voice PCs and their significance are shown in Table 2. PC1 (OR = 0.58, P<0.0001), PC6 (OR = 1.57, P<0.001), PC17 (OR = 1.53, P<0.0001) and PC24 (OR = 1.45, P<0.05) significantly predicted depression.

**Table 2:** Binary logistic regression model of voice data.

	*β*	SE	*Wald*	*corrected P*	*OR*
**PC1**	-0.5486592	0.1007107	-5.448	7.85E-06***	0.58
**PC6**	0.4536389	0.1117325	4.06	7.56E-03**	1.57
**PC17**	0.4251865	0.093759	4.535	8.87E-04***	1.53
**PC24**	0.3687606	0.0984286	3.746	2.76E-02*	1.45

*P*<0.05*,

*P*<0.01**,

*P*<0.001***;

Bonferroni correction

Demographic data and voice data together explained 39.98% (*Nagelkerke's R*^*2*^) of the risk for depression. This indicate that the unique contribution of voice features is 29.11%. The *OR*s for the PCs and demographic variables included in the model are shown in Table 3; the demographic variables occupation (*OR* = 1.21, *P*<0.05), and voice features PC1 (*OR* = 0.59, *P*<0.001), and PC17 (*OR* = 1.54, *P*<0.01) significantly predicted depression.

**Table 3:** Binary logistic regression model of demographic data and voice data.

	*β*	SE	*Wald*	corrected *P*	OR
**occupation**	0.19	0.05	3.82	0.021*	1.21
**PC1**	-0.53	0.12	-4.54	8.65E-04***	0.59
**PC17**	0.43	0.10	4.35	2.1E-03**	1.54

*P*<0.05*,

*P*<0.01**,

*P*<0.001***;

Bonferroni correction

More importantly, we compared model fitness between different input. See Tables 4 and 5. In Table 4, we compare model fitness based on only voice data and demographic data + voice data. Results showed that model fitness improved when voice data entered the model(ϰ^2^ = -241.11, P<0.001). In Table 5, model fitness between models on only voice data and only demographic data showed that model fitness of voice data is significantly better than demographic data(ϰ^2^ = -200.97, P<0.001).

**Table 4:** Compare model fitness between demo+voi and demo.

	resid.df	resid.dev	df	ϰ^2^	*P*
demo+voi^a^	764	886.59		-241.11	9.848E-08***
demo	901	1127.7	-137		

^a^ demo:demographic data; voi: voice data

*P*<0.001***

**Table 5:** Compare model fitness between demo+voi and demo.

	resid.df	resid.dev	df	ϰ^2^	*P*
voi	780	926.73		-200.97	6.77E-06***
demo	901	1127.7	-121		

*P*<0.001***

#### Classification modeling

Classification models were constructed to test whether voice features could be used to diagnose depression. We used feature selection, a method that improves a model’s generalizability and avoids the curse of dimensionality. We identified 37 features (1 demographic variable and 36 voice features). These features are listed in S2 Table. Please check in the *Supporting Information*.

All classification models were built based on these 37 features. The training set was from the 70% of D2.A. An *F measure* was adopted to estimate to what extent models correctly classified depression patients and the controls.

#### Testing model performance on rest 30% of D2.A

When predictions were obtained for the test set of D2.A. Results showed, when only demographic data was used to classify depression, the classification accuracy (*F measure*) was 73%. When only voice data was in the classification model, the accuracy was 81%. While the classification accuracy of demographic data and voice data is 81%. Compared to the classification accuracy of models based on demographic data alone, the classification accuracy of the models with voice data is higher. These results are shown in Table 6.

**Table 6:** Classification results for the test set from D2.A.

	*Accuracy*	*Precision*	*Recall*	*F-measure*
**demo**	0.66	0.76	0.72	0.73
**voi**	0.72	0.73	0.91	**0.81**
**demo+voi**	0.73	0.75	0.87	0.81

#### Testing model performance on D2.B

The number of participants overlap between D2.A and D2.B is 410. When predictions were obtained for the test set of D2.B. Results showed, when only demographic data was used to classify depression, the classification accuracy (*F measure*) was 75%. When only voice data was in the classification model, the accuracy was 80%. While the classification accuracy of demographic data and voice data is 81%. Compared to the classification accuracy of models based on demographic data alone, the classification accuracy of the models with voice data is consistently higher. These results are shown in Table 7.

**Table 7:** Classification results for the test set from D2.B.

	*accuracy*	*precision*	*recall*	*F-measure*
**demo**	0.67	0.78	0.73	0.75
**voi**	0.71	0.77	0.84	**0.8**
**demo+voi**	0.73	0.8	0.81	0.81

#### Testing model performance on 973 dataset

To further test model robustness across different emotion context, we tested our model of 70% D2.A training set on three different emotion value relevant dataset under 973 data.

In the positive emotion situation, results showed, when only demographic data was used to classify depression, the classification accuracy (*F measure*) was 69%. When only voice data was in the classification model, the accuracy was 75%. While the classification accuracy of demographic data and voice data is 77%. Compared to the classification accuracy of models based on demographic data alone, the classification accuracy of the models with voice data is also consistently higher. These results are shown in Table 8.

**Table 8:** Classification results for the test set from 973 data under positive emotion context.

	*accuracy*	*precision*	*recall*	*F-measure*
**demo**	0.62	0.76	0.64	0.69
**voi**	0.63	0.69	0.82	**0.75**
**demo+voi**	0.66	0.7	0.85	0.77

In the neural emotion situation, results showed, when only demographic data was used to classify depression, the classification accuracy (*F measure*) was 75%. When only voice data was in the classification model, the accuracy was 80%. While the classification accuracy of demographic data and voice data is 81%. Compared to the classification accuracy of models based on demographic data alone, the classification accuracy of the models with voice data is higher, consistent to former results. These results are shown in Table 9.

**Table 9:** Classification results for the test set from 973 data under neutral emotion context.

	*accuracy*	*precision*	*recall*	*F-measure*
**demo**	0.67	0.78	0.73	0.75
**voi**	0.71	0.77	0.84	**0.8**
**demo+voi**	0.73	0.8	0.81	0.81

In the negative emotion situation, results showed, when only demographic data was used to classify depression, the classification accuracy (*F measure*) was 69%. When only voice data was in the classification model, the accuracy was 76%. While the classification accuracy of demographic data and voice data is 78%. Compared to the classification accuracy of models based on demographic data alone, the classification accuracy of the models with voice data is also higher, consistent to former results. These results are shown in Table 10.

**Table 10:** Classification results for the test set from 973 data under negative emotion context.

	*accuracy*	*precision*	*recall*	*F-measure*
**demo**	0.62	0.76	0.64	0.69
**voi**	0.64	0.68	0.87	**0.76**
**demo+voi**	0.66	0.7	0.87	0.78

## Discussion

In this study, the relationships among voice features, demographic variables, and depression were systematically examined. Our findings addressed the primary goals of the study finding that (1) voice features make a significant contribution to the prediction of depression; (2) In regression model, the model fitness of voice data alone is significantly better than the model fitness of demographic data alone; (3) Depression classification models built on voice features are effective with generalization ability; (4) Depression classification models built on voice features are robust across different emotion situations.

First, binary logistic regression analysis showed that voice features significantly contribute to the prediction of depression (voice features, PC1, PC6, PC1, and PC24, *P*<0.05). What is more, when voice data and demographic data was included, compared to the results of only demographic data in the model, the variance in depression explained by voice data was 29.11%, which represents the unique contribution of the voice features. What’s more, the model fitness of voice data alone is significantly higher than that of demographic data, and when add voice data in the the demographic data based model, model fitness is also significantly improved.

This is the first time to systematically investigate whether voice features significantly predict depression, with a baseline of demographic variables. Several studies have investigated the correlation between voice features, such as prosodic and cepstral features, and depression [17,18,20]. Our results are the first to confirm that voice features can significantly predict depression, with considerable amount of contribution. There is no direct theory that specifies the mechanism of voice features in predicting depression; however, there is evidence to support our results.

On one hand, depression is associated with sustained activity in the brain areas responsible for coding emotional information [46,47]. On the other, plenty of studies have shown that voice parameters are affected by emotion. A review of the literature on human vocal emotion [48] noted that emotion influences voice in three main ways—voice quality, utterance timing, and utterance pitch contour. It has been suggested that basic emotions have a stable effect on voice features across different cultural backgrounds [48–50]. Prosodic features have been considered to be the most important factor in emotion recognition [51]. Spectrum features are also important in conveying emotion [52,53]. These findings show that depression patients may have stable emotional changes and corresponding speaking characteristics, which calls for further examination.

Second, the classification results of our study are in agreement: the classification accuracy of voice features is consistently higher than demographic data in each testing situation. More importantly, voice data can be used to predict depression under different emotion status, meaning depression detection using voice features is reliable and has its potential in clinical situation. This also indicate that voice features is a stable feature of depression, despite their emotion changes. The classification accuracy of voice features alone reached 81%(see Table 7). Classification models based on voice features alone have been widely examined. Classifying accuracy have been reported as between 60% and 90% in the machine learning field [17–21,28,29]. Here, the voice features following feature selection in the classification modeling were mainly calculated using some basic physical voice features: loudness, MFCC, LSP, voicing probability Both MFCCs and LSP are spectral features [54]; loudness, and voicing probability are prosodic features [55].

Spectral features, particularly MFCCs, were useful in classifying depression or not with an accuracy of 80% [18]. Spectral features are believed to reflect the relationship between changes in vocal tract shape and articulator movements [56]. These features have been observed to change in relation to the mental state of the speaker, relating to changes in muscle tension and control [16].

Prosodic features are the properties of syllables and larger units of speech, which contribute to linguistic functions such as intonation, tone, stress, and rhythm [57]. Previous research has shown consistent results that indicate some prosodic abnormality in the voice of depression patients. To illustrate, listeners were able to sense change in pitch, volume, speech rate, and pronunciation before and after treatment [58]. Prosody reflects various features of the speaker or the utterance, including: the emotional state of the speaker; the form of the utterance (statement, question, or command); the presence of irony or sarcasm; emphasis, contrast), and focus); and other elements of language that may not be encoded by grammar or by choice of vocabulary [59].

Taken together, our research has replicated previous results in which voice features were found to classify depression and has shown a stable generalizability when applied to new datasets, even under different emotion context. Though the length of voice recordings in our research are around10s, research on the same interview speech dataset has showed even 10 seconds length can reach ideal classification accuracy [60]. What’s more, short utterance has been proved to be effective in speaker identification [61–64]. The consistently higher accuracy in this research also showed short voice recordings can reach ideal predicting accuracy.

In addition, demographic variables also significantly predicted depression (see Tables 1 and 3) and showed great predictive accuracy when classifying depression (see Tables 6,7, 8, 9 and 10). These results indicate that demographic information can improve the classification accuracy of depression in clinical applications, which should be noted by further research.

There are also some limitations in this research. First, our study only includes females, which means we should be more careful when generalize our conclusions to the male population, the intention of only females included is to keep both high sample homogeneity and large sample size, besides voice features between males and females differs a lot. Second, there exists bias on demographic variables, which affected the reliability to some degree. But we have to admit this is part of the nature of observational study, especially for epidemiological studies. And we tried our best to control the effect of confounders. This may implied that there exist some pattern between depression and demographic variables. And also remind us the necessarity to examine and control the effect of demographic variables.

Despite its shortcomings, this study took a pioneering step in examining the predictive effect of voice features with consideration of confounding factors. Demographic characteristics, such as gender, age, emotion, or personality of the speaker, etc., have been shown to be strong confounding factors for depression detection systems [18,25,26] Most of these studies did not investigate the effect of these factors in a comprehensive way [19,20,28,29]. Morales et al. [23] mentioned the importance of examining and controlling the effect of confounding factors. Sex-independent models have been built [19]. Despite these studies, there has been no such work on evaluating the predicting effect of voice data with comparing baseline of potential confounding factors and how confounding factors affect the occurrence of depression.

## Conclusion

Taken together, our findings suggest that voice features play an important role in predicting depression. In addition, demographic variables should be valued in future research. Our results contribute to our understanding of the actual effect of voice features on depression. This research provides a foundation to further explore more robust classification models, as well as to identify related voice features to build more robust models and exploit the clinical application value of voice features to the fullest.

## Supporting information

S1 Table Table containing data description information. (DOCX)

S2 Table Features selected for model building. (DOCX)

S1 Appendix Voice dataset for answer recordings to question D2.A. (CSV)

S2 Appendix Voice dataset for answer recordings to question D2.B. (CSV)

S3 Appendix Voice dataset for recordings in 973 dataset. (CSV)
