# Supplementary material for: Re-examining the robustness of voice features in predicting depression: Compared with baseline of confounders
Source: PLoS One. 2019 Jun 20;14(6):e0218172. doi: 10.1371/journal.pone.0218172 (PMC6586278; doi:10.1371/journal.pone.0218172)
Supplement: S1 Table — (DOCX) [file pone.0218172.s001.docx]

**S1 Table. Data Description**

**Part a: before matching**

- **Demographic information of D2.A**

| Age | | | | |
| --- | --- | --- | --- | --- |
|  | ***M*** | ***sd*** | *z* | *P* |
| **control** | 47.34 | 5.40 | 4.86 | <0.000*** |
| **case** | 44.89 | 8.75 |  |  |

| **Occupation** | | | | | | | |
| --- | --- | --- | --- | --- | --- | --- | --- |
| **iscase** | **1** | **2** | **3** | **4** | **5** | ***ϰ^2^*** | ***P*** |
| 0 | 280 | 15 | 94 | 106 | 53 | 90.4 | 0.000*** |
| 1 | 178 | 72 | 121 | 184 | 29 |  |  |

| **Education** | | | | | | | | | |
| --- | --- | --- | --- | --- | --- | --- | --- | --- | --- |
| **iscase** | **1** | **2** | **3** | **4** | **5** | **6** | **7** | ***ϰ^2^*** | ***P*** |
| 0 | 15 | 61 | 207 | 116 | 43 | 60 | 46 | 13.65 | 0.03* |
| 1 | 34 | 78 | 183 | 121 | 56 | 53 | 59 |  |  |

| **Marital.status** | | | | | | |
| --- | --- | --- | --- | --- | --- | --- |
| **iscase** | **1** | **2** | **3** | **4** | ***ϰ^2^*** | ***P*** |
| 0 | 511 | 5 | 20 | 12 | 20.89 | <0.000*** |
| 1 | 503 | 27 | 40 | 14 |  |  |

| **Social.class** | | | | | | | |
| --- | --- | --- | --- | --- | --- | --- | --- |
| **iscase** | **1** | **2** | **3** | **4** | **5** | ***ϰ^2^*** | ***P*** |
| **0** | 63.00 | 132.00 | 91 | 154.00 | 108.00 | 8.08 | 0.08 |
| **1** | 68.00 | 181.00 | 82 | 158.00 | 95.00 |  |  |

| **Accent** | | | | | | | | | | | | |
| --- | --- | --- | --- | --- | --- | --- | --- | --- | --- | --- | --- | --- |
|  | **beijing** | **dongbei** | **jianghuai** | **jiaoliao** | **jilu** | **jin** | **lanyin** | **min** | **wu** | **xinan** | **yue** | **zhongyuan** |
| 0 | 7 | 105 | 108 | 13 | 36 | 45 | 24 | 7 | 50 | 15 | 17 | 121 |
| 1 | 10 | 98 | 40 | 21 | 53 | 31 | 16 | 8 | 135 | 30 | 30 | 112 |
| ϰ^2^ | **0.36** | 1.09 | 41.13 | 1.45 | 2.45 | 3.81 | 2.23 | 0.02 | 40.49 | 4.27 | 2.94 | 1.46 |
| P | **0.64** | 0.31 | <0.000*** | 0.29 | 0.12 | 0.06 | 0.15 | 1 | <0.000*** | 0.05 | 0.1 | 0.24 |

- **Demographic information of D2.B**

| **Age** | | | | |
| --- | --- | --- | --- | --- |
|  | ***M*** | ***sd*** | ***z*** | ***P*** |
| **control** | 47.15 | 5.99 | 3.52 | 0.001** |
| **case** | 44.89 | 8.74 |  |  |

| **Occupation** | | | | | | | |
| --- | --- | --- | --- | --- | --- | --- | --- |
| **iscase** | **1** | **2** | **3** | **4** | **5** | ***ϰ^2^*** | ***P*** |
| 0 | 98 | 8 | 42 | 64 | 17 | 23.48 | 0.000*** |
| 1 | 144 | 60 | 97 | 168 | 31 |  |  |

| **Education** | | | | | | | | | |
| --- | --- | --- | --- | --- | --- | --- | --- | --- | --- |
| **iscase** | **1** | **2** | **3** | **4** | **5** | **6** | **7** | ***ϰ^2^*** | ***P*** |
| 0 | 7 | 32 | 87 | 43 | 19 | 26 | 15 | 14.18 | 0.03* |
| 1 | 34 | 89 | 145 | 101 | 52 | 36 | 43 |  |  |

| **Marital.status** | | | | | |
| --- | --- | --- | --- | --- | --- |
| **iscase** | **1** | **3** | **4** | ***ϰ^2^*** | ***P*** |
| 0 | 213 | 11 | 5 | 3.25 | 0.21 |
| 1 | 450 | 42 | 8 |  |  |

| **Social.class** | | | | | | | |
| --- | --- | --- | --- | --- | --- | --- | --- |
| **iscase** | **1** | **2** | **3** | **4** | **5** | ***ϰ^2^*** | ***P*** |
| 0 | 22 | 54 | 41 | 66 | 46 | 3.41 | 0.49 |
| 1 | 47 | 148 | 74 | 141 | 90 |  |  |

| **Accent** | | | | | | | |
| --- | --- | --- | --- | --- | --- | --- | --- |
|  | **dongbei** | **jianghuai** | **jilu** | **lanyin** | **wu** | **xinan** | **zhongyuan** |
| 0 | 51 | 25 | 24 | 10 | 13 | 7 | 95 |
| 1 | 103 | 32 | 73 | 14 | 120 | 24 | 115 |
| ϰ^2^ | 0.26 | 4.45 | 2.31 | 1.21 | 35.35 | 1.17 | 26.17 |
| P | 0.63 | 0.04* | 0.16 | 0.37 | <0.000*** | 0.32 | <0.000*** |

- **Demographic information from 973 project**
- **Demographic information under positive emotion context**

| **Age** | | | | |
| --- | --- | --- | --- | --- |
|  | ***M*** | ***sd*** | ***z*** | ***P*** |
| **control** | 40.97 | 7.29 | -3.83 | 0.000*** |
| **case** | 43.62 | 8.01 |  |  |

| **Occupation** | | | | | | |
| --- | --- | --- | --- | --- | --- | --- |
| **iscase** | **1** | **3** | **4** | **5** | ***ϰ^2^*** | ***P*** |
| 0 | 189 | 7 | 7 | 35 | 19.93 | 0.000*** |
| 1 | 168 | 14 | 21 | 70 |  |  |

| **Education** | | | | | | | | |
| --- | --- | --- | --- | --- | --- | --- | --- | --- |
| **iscase** | **2** | **3** | **4** | **6** | **7** | **8** | ***ϰ^2^*** | ***P*** |
| 0 | 28 | 42 | 77 | 28 | 28 | 35 | 48.82 | <0.000*** |
| 1 | 42 | 49 | 56 | 42 | 77 | 7 |  |  |

- **Demographic information under neutral emotion context**

| **Age** | | | | |
| --- | --- | --- | --- | --- |
|  | ***M*** | ***sd*** | ***z*** | ***P*** |
| **control** | 40.97 | 7.29 | -3.83 | <0.000*** |
| **case** | 43.62 | 8.01 |  |  |

| **Occupation** | | | | | | |
| --- | --- | --- | --- | --- | --- | --- |
| **iscase** | **1** | **3** | **4** | **5** | ***ϰ^2^*** | ***P*** |
| 0 | 189 | 7 | 7 | 35 | 19.93 | 0.000*** |
| 1 | 168 | 14 | 21 | 70 |  |  |

| **Education** | | | | | | | | |
| --- | --- | --- | --- | --- | --- | --- | --- | --- |
| **iscase** | **2** | **3** | **4** | **6** | **7** | **8** | ***ϰ^2^*** | ***P*** |
| 0 | 28 | 42 | 77 | 28 | 28 | 35 | 48.82 | <0.000*** |
| 1 | 42 | 49 | 56 | 42 | 77 | 7 |  |  |

- **Demographic information under negative emotion context**

| **Age** | | | | |
| --- | --- | --- | --- | --- |
|  | **M** | **sd** | ***z*** | ***P*** |
| **control** | 40.97 | 7.29 | -3.83 | 0.000*** |
| **case** | 43.62 | 8.01 |  |  |

| **Occupation** | | | | | | |
| --- | --- | --- | --- | --- | --- | --- |
| **iscase** | **1** | **3** | **4** | **5** | ***ϰ^2^*** | ***P*** |
| 0 | 189 | 7 | 7 | 35 | 19.93 | 0.000*** |
| 1 | 168 | 14 | 21 | 70 |  |  |

| **Education** | | | | | | | | |
| --- | --- | --- | --- | --- | --- | --- | --- | --- |
| **iscase** | **2** | **3** | **4** | **6** | **7** | **8** | ***ϰ^2^*** | ***P*** |
| 0 | 28 | 42 | 77 | 28 | 28 | 35 | 48.82 | 0.000*** |
| 1 | 42 | 49 | 56 | 42 | 77 | 7 |  |  |

**Part b: Data description after matching**

- **Demographic information from D2.A**

| Age | | | | |
| --- | --- | --- | --- | --- |
|  | ***M*** | ***sd*** | ***z*** | ***P*** |
| **control** | 46.97 | 5.67 | 4.17 | <0.000*** |
| **case** | 44.70 | 8.84 |  |  |

| **Occupation** | | | | | | | |
| --- | --- | --- | --- | --- | --- | --- | --- |
| **iscase** | **1** | **2** | **3** | **4** | **5** | ***ϰ^2^*** | ***P*** |
| **0** | 151 | 11 | 68 | 85 | 19 | 34.94 | <0.000*** |
| **1** | 178 | 72 | 121 | 184 | 29 |  |  |

| **Education** | | | | | | | | | |
| --- | --- | --- | --- | --- | --- | --- | --- | --- | --- |
| **iscase** | **1** | **2** | **3** | **4** | **5** | **6** | **7** | ***ϰ^2^*** | ***P*** |
| 0 | 15 | 61 | 207 | 116 | 43 | 60 | 46 | 13.65 | 0.03 |
| 1 | 34 | 78 | 183 | 121 | 56 | 53 | 59 |  |  |

| **Marital.status** | | | | | | |
| --- | --- | --- | --- | --- | --- | --- |
| **iscase** | **1** | **2** | **3** | **4** | ***z*** | ***P*** |
| **0** | 309.00 | 5.00 | **13** | 7.00 | 10.24 | 0.01** |
| **1** | 503.00 | 27.00 | **40** | 14.00 |  |  |

| **Social.class** | | | | | | | |
| --- | --- | --- | --- | --- | --- | --- | --- |
| **iscase** | **1** | **2** | **3** | **4** | **5** | ***z*** | ***P*** |
| **0** | 39.00 | 70.00 | **54** | 110.00 | 61.00 | 11.49 | 0.02* |
| **1** | 68.00 | 181.00 | **82** | 158.00 | 95.00 |  |  |

| **Accent** | | | | | | | | | | | | |
| --- | --- | --- | --- | --- | --- | --- | --- | --- | --- | --- | --- | --- |
|  | **beijing** | **dongbei** | **jianghuai** | **jiaoliao** | **jilu** | **jin** | **lanyin** | **min** | **wu** | **xinan** | **yue** | **zhongyuan** |
| 0 | 6 | 66 | 28 | 9 | 30 | 28 | 9 | 6 | 36 | 11 | 14 | 91 |
| 1 | 10 | 98 | 40 | 21 | 53 | 31 | 16 | 8 | 135 | 30 | 30 | 112 |
| ϰ^2^ | **0.01** | 1.29 | 0.73 | 0.55 | 0 | 3.34 | 0 | 0.26 | 21.34 | 1.69 | 0.42 | 8.03 |
| P | **1** | 0.28 | 0.43 | 0.57 | 1 | 0.07 | 1 | 0.78 | <0.000*** | 0.24 | 0.53 | 0.01** |

- **Demographic information from D2.B**

| **Age** | | | | |
| --- | --- | --- | --- | --- |
|  | ***M*** | ***sd*** | ***z*** | ***P*** |
| **control** | 47.15 | 5.99 | 3.52 | 0.001** |
| **case** | 44.89 | 8.74 |  |  |

| **Occupation** | | | | | | | |
| --- | --- | --- | --- | --- | --- | --- | --- |
| **iscase** | **1** | **2** | **3** | **4** | **5** | ***ϰ^2^*** | ***P*** |
| 0 | 98 | 8 | 42 | 64 | 17 | 23.48 | 0.000*** |
| 1 | 144 | 60 | 97 | 168 | 31 |  |  |

| **Education** | | | | | | | | | |
| --- | --- | --- | --- | --- | --- | --- | --- | --- | --- |
| **iscase** | **1** | **2** | **3** | **4** | **5** | **6** | **7** | ***ϰ^2^*** | ***P*** |
| 0 | 7 | 32 | 87 | 43 | 19 | 26 | 15 | 14.18 | 0.03* |
| 1 | 34 | 89 | 145 | 101 | 52 | 36 | 43 |  |  |

| **marital.status** | | | | | |
| --- | --- | --- | --- | --- | --- |
| **iscase** | **1** | **3** | **4** | ***ϰ^2^*** | ***P*** |
| 0 | 213 | 11 | 5 | 3.25 | 0.21 |
| 1 | 450 | 42 | 8 |  |  |

| **Social.class** | | | | | | | |
| --- | --- | --- | --- | --- | --- | --- | --- |
| **iscase** | **1** | **2** | **3** | **4** | **5** | ***ϰ^2^*** | ***P*** |
| 0 | 22 | 54 | 41 | 66 | 46 | 3.41 | 0.49 |
| 1 | 47 | 148 | 74 | 141 | 90 |  |  |

| Accent | | | | | | | |
| --- | --- | --- | --- | --- | --- | --- | --- |
|  | **dongbei** | **jianghuai** | **jilu** | **lanyin** | **wu** | **xinan** | **zhongyuan** |
| 0 | 51 | 25 | 24 | 10 | 13 | 7 | 95 |
| 1 | 103 | 32 | 73 | 14 | 120 | 24 | 115 |
| ϰ^2^ | 0.26 | 4.45 | 2.31 | 1.21 | 35.35 | 1.17 | 26.17 |
| P | 0.63 | 0.04* | 0.16 | 0.37 | <0.000*** | 0.32 | <0.000*** |

- **Demographic information from 973 project**
- **Demographic information under positive emotion context**

| **Age** | | | | |
| --- | --- | --- | --- | --- |
|  | ***M*** | ***sd*** | ***z*** | ***P*** |
| **control** | 43.53 | 6.77 | -0.11 | 0.92 |
| **case** | 43.62 | 8.01 |  |  |

| **Occupation** | | | | | | |
| --- | --- | --- | --- | --- | --- | --- |
| **iscase** | **1** | **3** | **4** | **5** | ***ϰ^2^*** | ***P*** |
| 0 | 98 | 7 | 0 | 28 | 13.03 | 0.00** |
| 1 | 168 | 14 | 21 | 70 |  |  |

| **Education** | | | | | | | | |
| --- | --- | --- | --- | --- | --- | --- | --- | --- |
| **iscase** | **2** | **3** | **4** | **6** | **7** | **8** | ***ϰ^2^*** | ***P*** |
| 0 | 14 | 21 | 42 | 21 | 21 | 14 | 22.99 | 0.000*** |
| 1 | 42 | 49 | 56 | 42 | 77 | 7 |  |  |

- **Demographic information under neutral emotion context**

| **Age** | | | | |
| --- | --- | --- | --- | --- |
|  | ***M*** | ***sd*** | ***z*** | ***P*** |
| **control** | 43.53 | 6.77 | -0.11 | 0.92 |
| **case** | 43.62 | 8.01 |  |  |

| **Occupation** | | | | | | |
| --- | --- | --- | --- | --- | --- | --- |
| **iscase** | **1** | **3** | **4** | **5** | ***ϰ^2^*** | ***P*** |
| 0 | 98 | 7 | 0 | 28 | 13.03 | 0.00** |
| 1 | 168 | 14 | 21 | 70 |  |  |

| **Education** | | | | | | | | |
| --- | --- | --- | --- | --- | --- | --- | --- | --- |
| **iscase** | **2** | **3** | **4** | **6** | **7** | **8** | ***ϰ^2^*** | ***P*** |
| 0 | 14 | 21 | 42 | 21 | 21 | 14 | 22.99 | 0.000*** |
| 1 | 42 | 49 | 56 | 42 | 77 | 7 |  |  |

- **Demographic information under negative emotion context**

| Age | | | | |
| --- | --- | --- | --- | --- |
|  | **M** | **sd** | ***z*** | ***P*** |
| **control** | 42.84 | 6.94 | -0.95 | 0.34 |
| **case** | 43.62 | 8.10 |  |  |

| **Occupation** | | | | | | |
| --- | --- | --- | --- | --- | --- | --- |
| **iscase** | **1** | **3** | **4** | **5** | ***ϰ^2^*** | ***P*** |
| 0 | 91 | 7 | 0 | 35 | 10.91 | 0.01** |
| 1 | 168 | 14 | 21 | 70 |  |  |

| **Education** | | | | | | | | |
| --- | --- | --- | --- | --- | --- | --- | --- | --- |
| **iscase** | **2** | **3** | **4** | **6** | **7** | **8** | ***ϰ^2^*** | ***P*** |
| 0 | 14 | 28 | 42 | 14 | 21 | 14 | 24.725 | 0.000*** |
| 1 | 42 | 49 | 56 | 42 | 77 | 7 |  |  |
