# Supplementary material for: Re-examining the robustness of voice features in predicting depression: Compared with baseline of confounders
Source: PLoS One. 2019 Jun 20;14(6):e0218172. doi: 10.1371/journal.pone.0218172 (PMC6586278; doi:10.1371/journal.pone.0218172)
Supplement: S2 Table — (DOCX) [file pone.0218172.s002.docx]

**S2 Table Features obtained by feature selection**

| age |
| --- |
| pcm_intensity_sma_quartile1 |
| pcm_loudness_sma_linregerrA |
| pcm_loudness_sma_stddev |
| pcm_loudness_sma_iqr2.3 |
| pcm_loudness_sma_iqr1.3 |
| mfcc_sma.1._max |
| mfcc_sma.2._max |
| mfcc_sma.2._amean |
| mfcc_sma.5._min |
| mfcc_sma.5._stddev |
| mfcc_sma.5._iqr1.2 |
| mfcc_sma.6._min |
| lspFreq_sma.3._amean |
| lspFreq_sma.3._quartile1 |
| lspFreq_sma.3._quartile2 |
| lspFreq_sma.3._quartile3 |
| lspFreq_sma.4._amean |
| lspFreq_sma.4._quartile1 |
| lspFreq_sma.4._quartile2 |
| lspFreq_sma.5._amean |
| lspFreq_sma.5._quartile1 |
| mfcc_sma_de.2._quartile3 |
| mfcc_sma_de.2._iqr1.2 |
| mfcc_sma_de.2._iqr1.3 |
| mfcc_sma_de.3._linregerrA |
| mfcc_sma_de.3._linregerrQ |
| mfcc_sma_de.3._stddev |
| mfcc_sma_de.5._linregerrA |
| mfcc_sma_de.5._linregerrQ |
| mfcc_sma_de.5._stddev |
| mfcc_sma_de.7._linregerrA |
| mfcc_sma_de.7._linregerrQ |
| mfcc_sma_de.7._stddev |
| voiceProb_sma_de_quartile1 |
| voiceProb_sma_de_iqr1.2 |
| voiceProb_sma_de_iqr1.3 |
